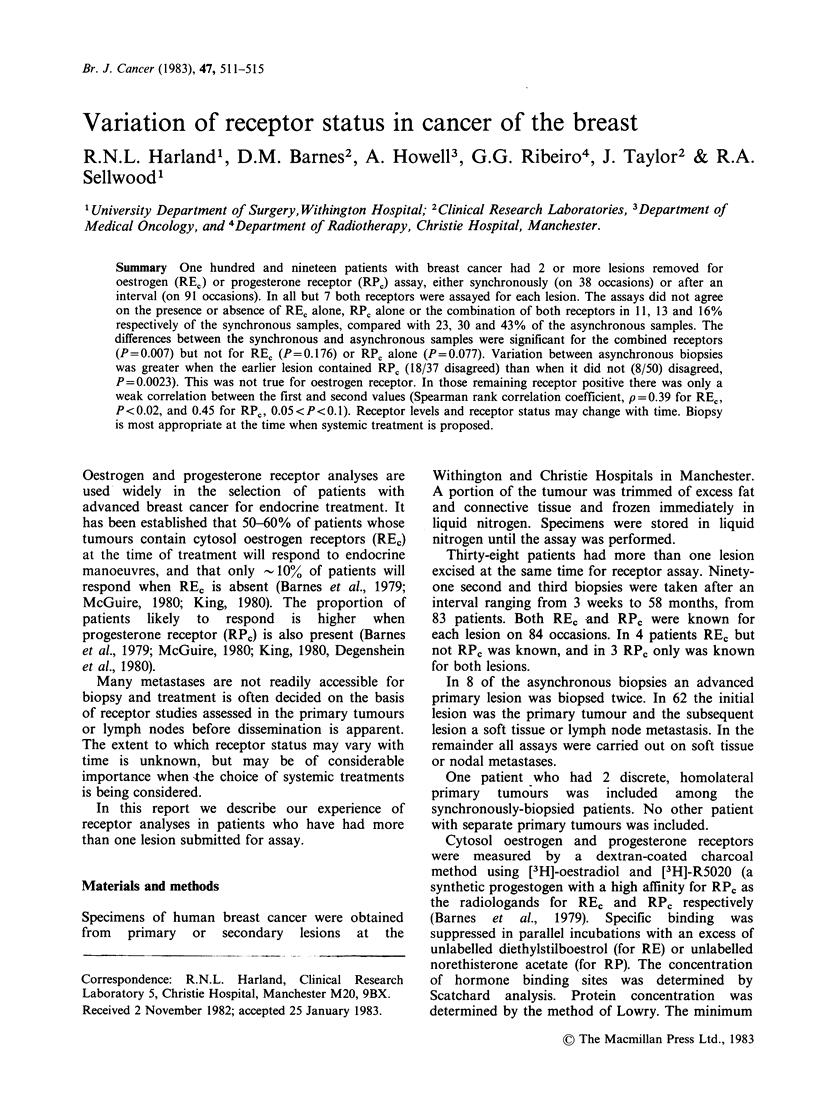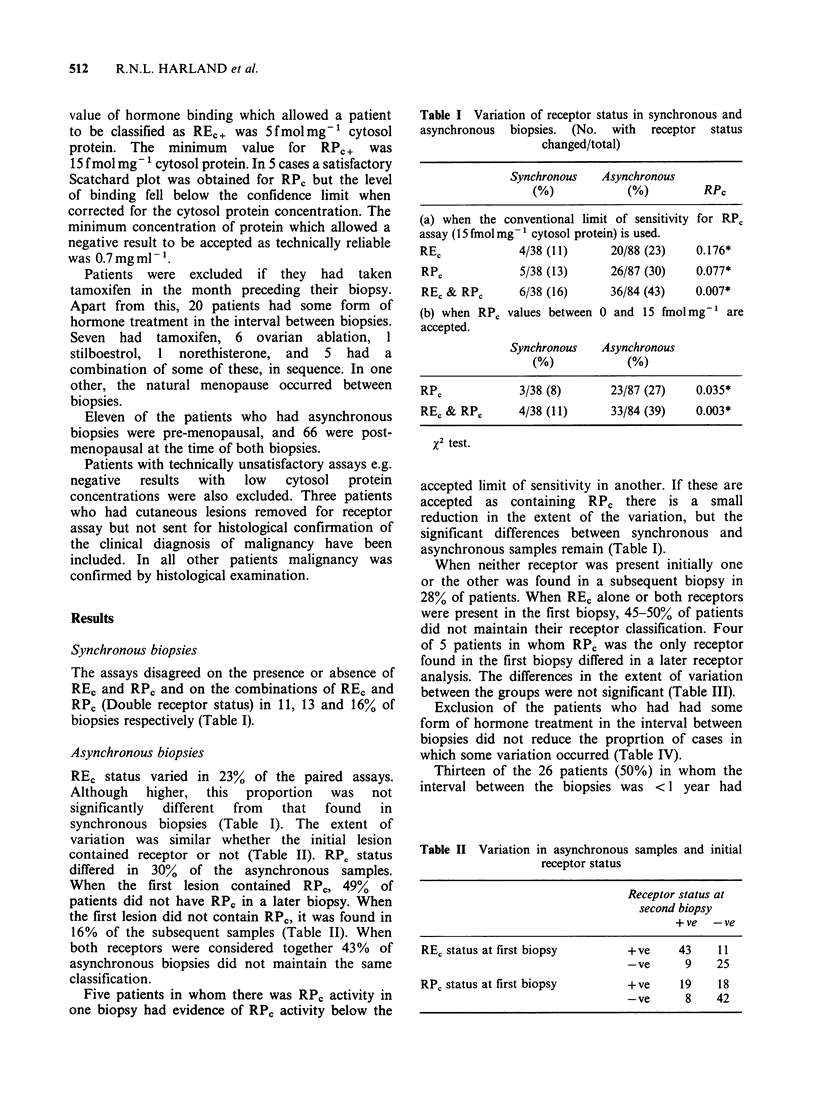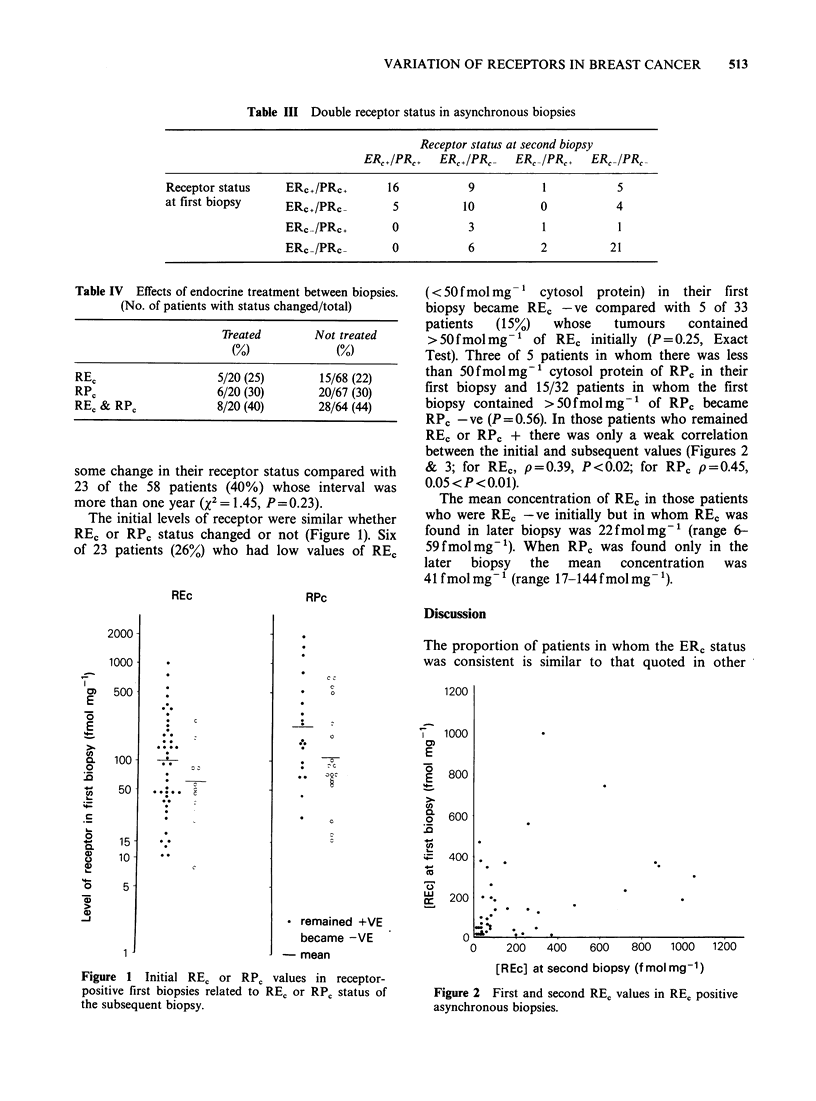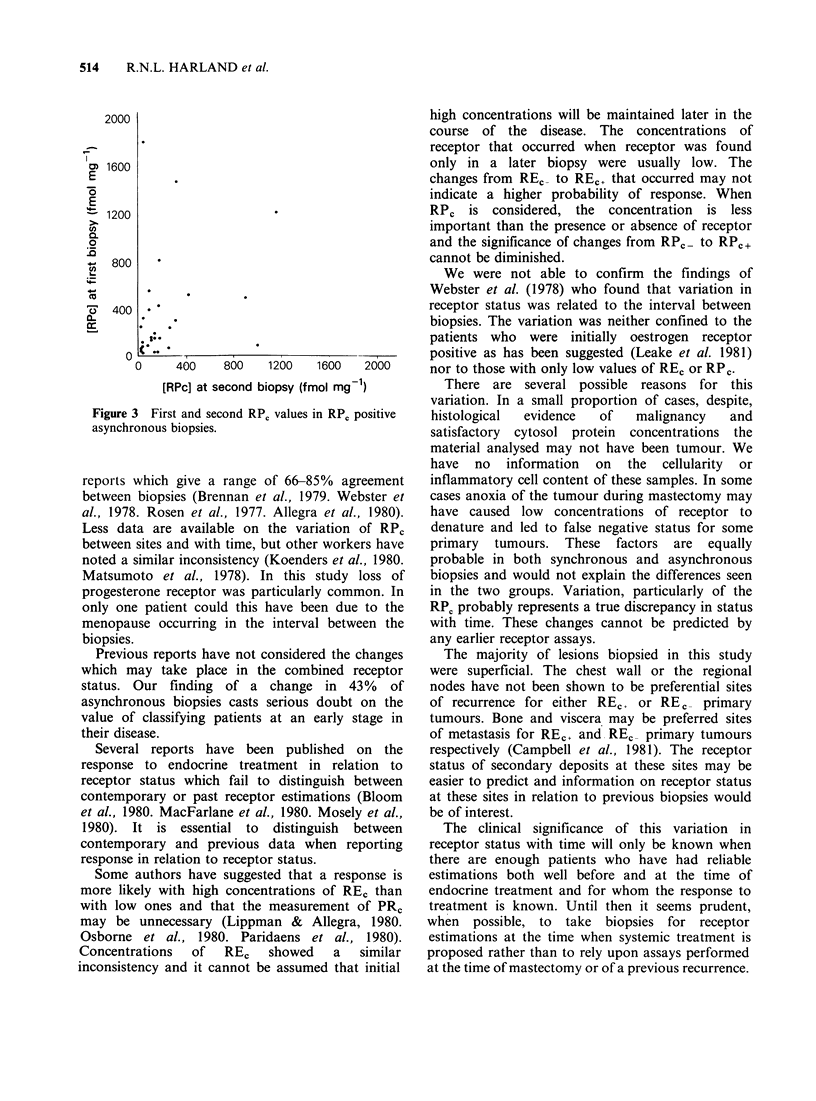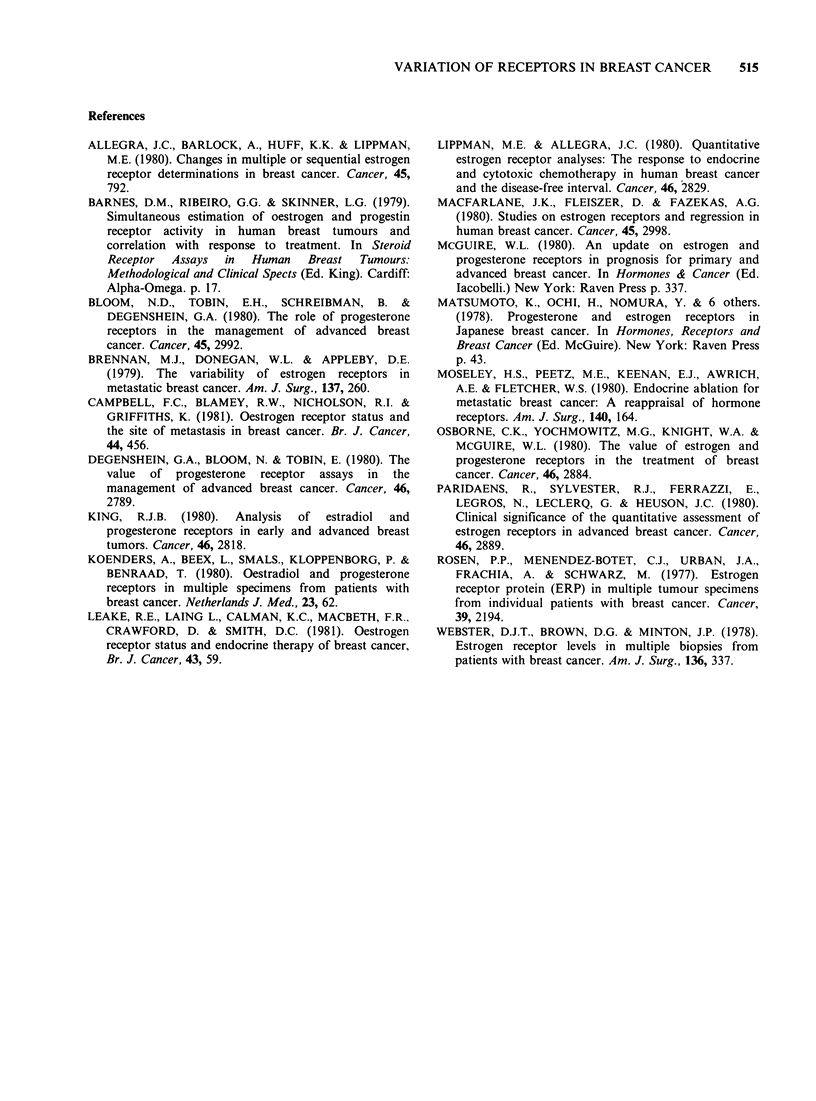# Variation of receptor status in cancer of the breast.

**DOI:** 10.1038/bjc.1983.81

**Published:** 1983-04

**Authors:** R. N. Harland, D. M. Barnes, A. Howell, G. G. Ribeiro, J. Taylor, R. A. Sellwood

## Abstract

One hundred and nineteen patients with breast cancer had 2 or more lesions removed for oestrogen (REc) or progesterone receptor (RPc) assay, either synchronously (on 38 occasions) or after an interval (on 91 occasions). In all but 7 both receptors were assayed for each lesion. The assays did not agree on the presence or absence of REc alone, RPc alone or the combination of both receptors in 11, 13 and 16% respectively of the synchronous samples, compared with 23, 30 and 43% of the asynchronous samples. The differences between the synchronous and asynchronous samples were significant for the combined receptors (P = 0.007) but not for REc (P = 0.176) or RPc alone (P = 0.077). Variation between asynchronous biopsies was greater when the earlier lesion contained RPc (18/37 disagreed) than when it did not (8/50) disagreed, P = 0.0023). This was not true for oestrogen receptor. In those remaining receptor positive there was only a weak correlation between the first and second values (Spearman rank correlation coefficient, rho = 0.39 for REc, P less than 0.02, and 0.45 for RPc, 0.05 less than P less than 0.1). Receptor levels and receptor status may change with time. Biopsy is most appropriate at the time when systemic treatment is proposed.


*Br. J. Cancer* (1983), **47**, 511–515

### Variation of receptor status in cancer of the breast

R.N.L. Harland[1], D.M. Barnes[2], A. Howell[3], G.G. Ribeiro[4], J. Taylor[2] & R.A. Sellwood[1]

[1]*University Department of Surgery, Withington Hospital;* [2]*Clinical Research Laboratories,* [3]*Department of Medical Oncology, and* [4]*Department of Radiotherapy, Christie Hospital, Manchester.*

**Summary** One hundred and nineteen patients with breast cancer had 2 or more lesions removed for oestrogen ($RE_c$) or progesterone receptor ($RP_c$) assay, either synchronously (on 38 occasions) or after an interval (on 91 occasions). In all but 7 both receptors were assayed for each lesion. The assays did not agree on the presence or absence of $RE_c$ alone, $RP_c$ alone or the combination of both receptors in 11, 13 and 16% respectively of the synchronous samples, compared with 23, 30 and 43% of the asynchronous samples. The differences between the synchronous and asynchronous samples were significant for the combined receptors ($P=0.007$) but not for $RE_c$ ($P=0.176$) or $RP_c$ alone ($P=0.077$). Variation between asynchronous biopsies was greater when the earlier lesion contained $RP_c$ (18/37 disagreed) than when it did not (8/50) disagreed, $P=0.0023$). This was not true for oestrogen receptor. In those remaining receptor positive there was only a weak correlation between the first and second values (Spearman rank correlation coefficient, $\rho=0.39$ for $RE_c$, $P<0.02$, and 0.45 for $RP_c$, $0.05<P<0.1$). Receptor levels and receptor status may change with time. Biopsy is most appropriate at the time when systemic treatment is proposed.

Oestrogen and progesterone receptor analyses are used widely in the selection of patients with advanced breast cancer for endocrine treatment. It has been established that 50–60% of patients whose tumours contain cytosol oestrogen receptors ($RE_c$) at the time of treatment will respond to endocrine manoeuvres, and that only ~10% of patients will respond when $RE_c$ is absent (Barnes *et al.*, 1979; McGuire, 1980; King, 1980). The proportion of patients likely to respond is higher when progesterone receptor ($RP_c$) is also present (Barnes *et al.*, 1979; McGuire, 1980; King, 1980, Degenshein *et al.*, 1980).

Many metastases are not readily accessible for biopsy and treatment is often decided on the basis of receptor studies assessed in the primary tumours or lymph nodes before dissemination is apparent. The extent to which receptor status may vary with time is unknown, but may be of considerable importance when the choice of systemic treatments is being considered.

In this report we describe our experience of receptor analyses in patients who have had more than one lesion submitted for assay.

### Materials and methods

Specimens of human breast cancer were obtained from primary or secondary lesions at the Withington and Christie Hospitals in Manchester. A portion of the tumour was trimmed of excess fat and connective tissue and frozen immediately in liquid nitrogen. Specimens were stored in liquid nitrogen until the assay was performed.

Thirty-eight patients had more than one lesion excised at the same time for receptor assay. Ninety-one second and third biopsies were taken after an interval ranging from 3 weeks to 58 months, from 83 patients. Both $RE_c$ and $RP_c$ were known for each lesion on 84 occasions. In 4 patients $RE_c$ but not $RP_c$ was known, and in 3 $RP_c$ only was known for both lesions.

In 8 of the asynchronous biopsies an advanced primary lesion was biopsed twice. In 62 the initial lesion was the primary tumour and the subsequent lesion a soft tissue or lymph node metastasis. In the remainder all assays were carried out on soft tissue or nodal metastases.

One patient who had 2 discrete, homolateral primary tumours was included among the synchronously-biopsied patients. No other patient with separate primary tumours was included.

Cytosol oestrogen and progesterone receptors were measured by a dextran-coated charcoal method using [$^3$H]-oestradiol and [$^3$H]-R5020 (a synthetic progestogen with a high affinity for $RP_c$ as the radiologands for $RE_c$ and $RP_c$ respectively (Barnes *et al.*, 1979). Specific binding was suppressed in parallel incubations with an excess of unlabelled diethylstilboestrol (for RE) or unlabelled norethisterone acetate (for RP). The concentration of hormone binding sites was determined by Scatchard analysis. Protein concentration was determined by the method of Lowry. The minimum

Correspondence: R.N.L. Harland, Clinical Research Laboratory 5, Christie Hospital, Manchester M20, 9BX.

Received 2 November 1982; accepted 25 January 1983.

© The Macmillan Press Ltd., 1983

value of hormone binding which allowed a patient to be classified as $RE_{c+}$ was $5\,\mathrm{fmol\,mg^{-1}}$ cytosol protein. The minimum value for $RP_{c+}$ was $15\,\mathrm{fmol\,mg^{-1}}$ cytosol protein. In 5 cases a satisfactory Scatchard plot was obtained for $RP_c$ but the level of binding fell below the confidence limit when corrected for the cytosol protein concentration. The minimum concentration of protein which allowed a negative result to be accepted as technically reliable was $0.7\,\mathrm{mg\,ml^{-1}}$.

Patients were excluded if they had taken tamoxifen in the month preceding their biopsy. Apart from this, 20 patients had some form of hormone treatment in the interval between biopsies. Seven had tamoxifen, 6 ovarian ablation, 1 stilboestrol, 1 norethisterone, and 5 had a combination of some of these, in sequence. In one other, the natural menopause occurred between biopsies.

Eleven of the patients who had asynchronous biopsies were pre-menopausal, and 66 were post-menopausal at the time of both biopsies.

Patients with technically unsatisfactory assays e.g. negative results with low cytosol protein concentrations were also excluded. Three patients who had cutaneous lesions removed for receptor assay but not sent for histological confirmation of the clinical diagnosis of malignancy have been included. In all other patients malignancy was confirmed by histological examination.

### Results

### *Synchronous biopsies*

The assays disagreed on the presence or absence of $RE_c$ and $RP_c$ and on the combinations of $RE_c$ and $RP_c$ (Double receptor status) in 11, 13 and 16% of biopsies respectively (Table I).

### *Asynchronous biopsies*

$RE_c$ status varied in 23% of the paired assays. Although higher, this proportion was not significantly different from that found in synchronous biopsies (Table I). The extent of variation was similar whether the initial lesion contained receptor or not (Table II). $RP_c$ status differed in 30% of the asynchronous samples. When the first lesion contained $RP_c$, 49% of patients did not have $RP_c$ in a later biopsy. When the first lesion did not contain $RP_c$, it was found in 16% of the subsequent samples (Table II). When both receptors were considered together 43% of asynchronous biopsies did not maintain the same classification.

Five patients in whom there was $RP_c$ activity in one biopsy had evidence of $RP_c$ activity below the accepted limit of sensitivity in another. If these are accepted as containing $RP_c$ there is a small reduction in the extent of the variation, but the significant differences between synchronous and asynchronous samples remain (Table I).

When neither receptor was present initially one or the other was found in a subsequent biopsy in 28% of patients. When $RE_c$ alone or both receptors were present in the first biopsy, 45–50% of patients did not maintain their receptor classification. Four of 5 patients in whom $RP_c$ was the only receptor found in the first biopsy differed in a later receptor analysis. The differences in the extent of variation between the groups were not significant (Table III).

Exclusion of the patients who had had some form of hormone treatment in the interval between biopsies did not reduce the proprtion of cases in which some variation occurred (Table IV).

Thirteen of the 26 patients (50%) in whom the interval between the biopsies was <1 year had

**Table I** Variation of receptor status in synchronous and asynchronous biopsies. (No. with receptor status changed/total)

| | *Synchronous* (%) | *Asynchronous* (%) | $RP_c$ |
|---|---|---|---|
| (a) when the conventional limit of sensitivity for $RP_c$ assay ($15\,\mathrm{fmol\,mg^{-1}}$ cytosol protein) is used. | | | |
| $RE_c$ | 4/38 (11) | 20/88 (23) | 0.176* |
| $RP_c$ | 5/38 (13) | 26/87 (30) | 0.077* |
| $RE_c$ & $RP_c$ | 6/38 (16) | 36/84 (43) | 0.007* |
| (b) when $RP_c$ values between 0 and 15 $\mathrm{fmol\,mg^{-1}}$ are accepted. | | | |
| | *Synchronous* (%) | *Asynchronous* (%) | |
| $RP_c$ | 3/38 (8) | 23/87 (27) | 0.035* |
| $RE_c$ & $RP_c$ | 4/38 (11) | 33/84 (39) | 0.003* |

$\chi^2$ test.

**Table II** Variation in asynchronous samples and initial receptor status

| | | *Receptor status at second biopsy* +*ve* | −*ve* |
|---|---|---|---|
| $RE_c$ status at first biopsy | +ve | 43 | 11 |
| | −ve | 9 | 25 |
| $RP_c$ status at first biopsy | +ve | 19 | 18 |
| | −ve | 8 | 42 |

**Table III** Double receptor status in asynchronous biopsies

| | | *Receptor status at second biopsy* | | | |
|---|---|---|---|---|---|
| | | $ER_{c+}/PR_{c+}$ | $ER_{c+}/PR_{c-}$ | $ER_{c-}/PR_{c+}$ | $ER_{c-}/PR_{c-}$ |
| Receptor status at first biopsy | $ER_{c+}/PR_{c+}$ | 16 | 9 | 1 | 5 |
| | $ER_{c+}/PR_{c-}$ | 5 | 10 | 0 | 4 |
| | $ER_{c-}/PR_{c+}$ | 0 | 3 | 1 | 1 |
| | $ER_{c-}/PR_{c-}$ | 0 | 6 | 2 | 21 |

**Table IV** Effects of endocrine treatment between biopsies. (No. of patients with status changed/total)

| | *Treated* (%) | *Not treated* (%) |
|---|---|---|
| $RE_c$ | 5/20 (25) | 15/68 (22) |
| $RP_c$ | 6/20 (30) | 20/67 (30) |
| $RE_c$ & $RP_c$ | 8/20 (40) | 28/64 (44) |

some change in their receptor status compared with 23 of the 58 patients (40%) whose interval was more than one year ($\chi^2 = 1.45$, $P = 0.23$).

The initial levels of receptor were similar whether $RE_c$ or $RP_c$ status changed or not (Figure 1). Six of 23 patients (26%) who had low values of $RE_c$ (<50 fmol mg$^{-1}$ cytosol protein) in their first biopsy became $RE_c$ −ve compared with 5 of 33 patients (15%) whose tumours contained >50 fmol mg$^{-1}$ of $RE_c$ initially ($P = 0.25$, Exact Test). Three of 5 patients in whom there was less than 50 fmol mg$^{-1}$ cytosol protein of $RP_c$ in their first biopsy and 15/32 patients in whom the first biopsy contained >50 fmol mg$^{-1}$ of $RP_c$ became $RP_c$ −ve ($P = 0.56$). In those patients who remained $RE_c$ or $RP_c$ + there was only a weak correlation between the initial and subsequent values (Figures 2 & 3; for $RE_c$, $\rho = 0.39$, $P < 0.02$; for $RP_c$ $\rho = 0.45$, $0.05 < P < 0.01$).

The mean concentration of $RE_c$ in those patients who were $RE_c$ −ve initially but in whom $RE_c$ was found in later biopsy was 22 fmol mg$^{-1}$ (range 6–59 fmol mg$^{-1}$). When $RP_c$ was found only in the later biopsy the mean concentration was 41 fmol mg$^{-1}$ (range 17–144 fmol mg$^{-1}$).

**Figure 1** Initial $RE_c$ or $RP_c$ values in receptor-positive first biopsies related to $RE_c$ or $RP_c$ status of the subsequent biopsy.

### Discussion

The proportion of patients in whom the $ER_c$ status was consistent is similar to that quoted in other

**Figure 2** First and second $RE_c$ values in $RE_c$ positive asynchronous biopsies.

**Figure 3** First and second $RP_c$ values in $RP_c$ positive asynchronous biopsies.

reports which give a range of 66–85% agreement between biopsies (Brennan *et al.*, 1979. Webster *et al.*, 1978. Rosen *et al.*, 1977. Allegra *et al.*, 1980). Less data are available on the variation of $RP_c$ between sites and with time, but other workers have noted a similar inconsistency (Koenders *et al.*, 1980. Matsumoto *et al.*, 1978). In this study loss of progesterone receptor was particularly common. In only one patient could this have been due to the menopause occurring in the interval between the biopsies.

Previous reports have not considered the changes which may take place in the combined receptor status. Our finding of a change in 43% of asynchronous biopsies casts serious doubt on the value of classifying patients at an early stage in their disease.

Several reports have been published on the response to endocrine treatment in relation to receptor status which fail to distinguish between contemporary or past receptor estimations (Bloom *et al.*, 1980. MacFarlane *et al.*, 1980. Mosely *et al.*, 1980). It is essential to distinguish between contemporary and previous data when reporting response in relation to receptor status.

Some authors have suggested that a response is more likely with high concentrations of $RE_c$ than with low ones and that the measurement of $PR_c$ may be unnecessary (Lippman & Allegra, 1980. Osborne *et al.*, 1980. Paridaens *et al.*, 1980). Concentrations of $RE_c$ showed a similar inconsistency and it cannot be assumed that initial high concentrations will be maintained later in the course of the disease. The concentrations of receptor that occurred when receptor was found only in a later biopsy were usually low. The changes from $RE_{c-}$ to $RE_{c+}$ that occurred may not indicate a higher probability of response. When $RP_c$ is considered, the concentration is less important than the presence or absence of receptor and the significance of changes from $RP_{c-}$ to $RP_{c+}$ cannot be diminished.

We were not able to confirm the findings of Webster *et al.* (1978) who found that variation in receptor status was related to the interval between biopsies. The variation was neither confined to the patients who were initially oestrogen receptor positive as has been suggested (Leake *et al.* 1981) nor to those with only low values of $RE_c$ or $RP_c$.

There are several possible reasons for this variation. In a small proportion of cases, despite, histological evidence of malignancy and satisfactory cytosol protein concentrations the material analysed may not have been tumour. We have no information on the cellularity or inflammatory cell content of these samples. In some cases anoxia of the tumour during mastectomy may have caused low concentrations of receptor to denature and led to false negative status for some primary tumours. These factors are equally probable in both synchronous and asynchronous biopsies and would not explain the differences seen in the two groups. Variation, particularly of the $RP_c$ probably represents a true discrepancy in status with time. These changes cannot be predicted by any earlier receptor assays.

The majority of lesions biopsied in this study were superficial. The chest wall or the regional nodes have not been shown to be preferential sites of recurrence for either $RE_{c+}$ or $RE_{c-}$ primary tumours. Bone and viscera may be preferred sites of metastasis for $RE_{c+}$ and $RE_{c-}$ primary tumours respectively (Campbell *et al.*, 1981). The receptor status of secondary deposits at these sites may be easier to predict and information on receptor status at these sites in relation to previous biopsies would be of interest.

The clinical significance of this variation in receptor status with time will only be known when there are enough patients who have had reliable estimations both well before and at the time of endocrine treatment and for whom the response to treatment is known. Until then it seems prudent, when possible, to take biopsies for receptor estimations at the time when systemic treatment is proposed rather than to rely upon assays performed at the time of mastectomy or of a previous recurrence.

### References

ALLEGRA, J.C., BARLOCK, A., HUFF, K.K. & LIPPMAN, M.E. (1980). Changes in multiple or sequential estrogen receptor determinations in breast cancer. *Cancer*, **45**, 792.

BARNES, D.M., RIBEIRO, G.G. & SKINNER, L.G. (1979). Simultaneous estimation of oestrogen and progestin receptor activity in human breast tumours and correlation with response to treatment. In *Steroid Receptor Assays in Human Breast Tumours: Methodological and Clinical Spects* (Ed. King). Cardiff: Alpha-Omega. p. 17.

BLOOM, N.D., TOBIN, E.H., SCHREIBMAN, B. & DEGENSHEIN, G.A. (1980). The role of progesterone receptors in the management of advanced breast cancer. *Cancer*, **45**, 2992.

BRENNAN, M.J., DONEGAN, W.L. & APPLEBY, D.E. (1979). The variability of estrogen receptors in metastatic breast cancer. *Am. J. Surg.*, **137**, 260.

CAMPBELL, F.C., BLAMEY, R.W., NICHOLSON, R.I. & GRIFFITHS, K. (1981). Oestrogen receptor status and the site of metastasis in breast cancer. *Br. J. Cancer*, **44**, 456.

DEGENSHEIN, G.A., BLOOM, N. & TOBIN, E. (1980). The value of progesterone receptor assays in the management of advanced breast cancer. *Cancer*, **46**, 2789.

KING, R.J.B. (1980). Analysis of estradiol and progesterone receptors in early and advanced breast tumors. *Cancer*, **46**, 2818.

KOENDERS, A., BEEX, L., SMALS., KLOPPENBORG, P. & BENRAAD, T. (1980). Oestradiol and progesterone receptors in multiple specimens from patients with breast cancer. *Netherlands J. Med.*, **23**, 62.

LEAKE, R.E., LAING L., CALMAN, K.C., MACBETH, F.R., CRAWFORD, D. & SMITH, D.C. (1981). Oestrogen receptor status and endocrine therapy of breast cancer, *Br. J. Cancer*, **43**, 59.

LIPPMAN, M.E. & ALLEGRA, J.C. (1980). Quantitative estrogen receptor analyses: The response to endocrine and cytotoxic chemotherapy in human breast cancer and the disease-free interval. *Cancer*, **46**, 2829.

MACFARLANE, J.K., FLEISZER, D. & FAZEKAS, A.G. (1980). Studies on estrogen receptors and regression in human breast cancer. *Cancer*, **45**, 2998.

MCGUIRE, W.L. (1980). An update on estrogen and progesterone receptors in prognosis for primary and advanced breast cancer. In *Hormones & Cancer* (Ed. Iacobelli.) New York: Raven Press p. 337.

MATSUMOTO, K., OCHI, H., NOMURA, Y. & 6 others. (1978). Progesterone and estrogen receptors in Japanese breast cancer. In *Hormones, Receptors and Breast Cancer* (Ed. McGuire). New York: Raven Press p. 43.

MOSELEY, H.S., PEETZ, M.E., KEENAN, E.J., AWRICH, A.E. & FLETCHER, W.S. (1980). Endocrine ablation for metastatic breast cancer: A reappraisal of hormone receptors. *Am. J. Surg.*, **140**, 164.

OSBORNE, C.K., YOCHMOWITZ, M.G., KNIGHT, W.A. & MCGUIRE, W.L. (1980). The value of estrogen and progesterone receptors in the treatment of breast cancer. *Cancer*, **46**, 2884.

PARIDAENS, R., SYLVESTER, R.J., FERRAZZI, E., LEGROS, N., LECLERQ, G. & HEUSON, J.C. (1980). Clinical significance of the quantitative assessment of estrogen receptors in advanced breast cancer. *Cancer*, **46**, 2889.

ROSEN, P.P., MENENDEZ-BOTET, C.J., URBAN, J.A., FRACHIA, A. & SCHWARZ, M. (1977). Estrogen receptor protein (ERP) in multiple tumour specimens from individual patients with breast cancer. *Cancer*, **39**, 2194.

WEBSTER, D.J.T., BROWN, D.G. & MINTON, J.P. (1978). Estrogen receptor levels in multiple biopsies from patients with breast cancer. *Am. J. Surg.*, **136**, 337.